# Birth Extraordinary

**Published:** 1865-02

**Authors:** 


Birth Extraordinary.—A few days since the wife of a loborer of Leamington gave birth to four children, all still-born.—*Lancet.*

